# Impact Of Prepregnancy Overweight And Obesity On Treatment Modality And Pregnancy Outcome In Women With Gestational Diabetes Mellitus

**DOI:** 10.3389/fendo.2022.799625

**Published:** 2022-05-19

**Authors:** Tina Linder, Anna Eder, Cécile Monod, Ingo Rosicky, Daniel Eppel, Katharina Redling, Franziska Geissler, Evelyn A. Huhn, Irene Hösli, Christian S. Göbl

**Affiliations:** ^1^Department of Obstetrics and Gynaecology, Division of Obstetrics and feto-maternal Medicine, Medical University of Vienna, Vienna, Austria; ^2^Department of Obstetrics and Gynaecology, University Hospital Basel, Basel, Switzerland

**Keywords:** gestational diabetes (GDM), obesity, pharmacotherapy, pregnancy outcome, birth weight

## Abstract

**Background:**

We aim to evaluate the impact of prepregnancy overweight on treatment modalities of Gestational Diabetes Mellitus (GDM). We assessed the association of increased pregravid Body Mass Index (BMI) with dosing of basal and rapid acting insulin as well as pregnancy outcome.

**Methods:**

We included 509 gestational diabetic women (normal weight: 200, overweight: 157, obese: 152), attending the pregnancy outpatient clinic at the Department of Obstetrics and Gynecology, Medical University of Vienna, in this retrospective study. We used a prospectively compiled database to assess patient characteristics, treatment approaches – particularly maximum doses of basal and rapid acting insulin or metformin – and pregnancy outcome.

**Results:**

Increased BMI was associated with the need of glucose lowering medication (odds ratio (OR): 1.08 for the increase of 1 kg/m² BMI, 95%CI 1.05–1.11, p<0.001). Mothers with pregestational obesity received the highest amount of insulin. Metformin was more often used in patients with obesity who also required higher daily doses. Maternal BMI was associated with increased risk of cesarean section (OR 1.04, 95%CI 1.01–1.07, p<0.001) and delivering large for gestational age offspring (OR 1.09, 95%CI 1.04–1.13, p<0.001). Birthweight percentiles were highest in patients with obesity who required glucose lowering therapy.

**Conclusions:**

Treatment modalities and outcome in GDM pregnancies are closely related to the extent of maternal BMI. Patients with obesity required glucose lowering medication more often and were at higher risk of adverse pregnancy outcomes. It is crucial to further explore the underlying pathophysiologic mechanisms to optimize clinical management and individual treatment approaches.

## Introduction

Growing evidence suggests that GDM comprises a broad spectrum of metabolic entities. It is crucial to identify factors which contribute to the observed heterogeneity in treatment modalities and outcomes of the disease (1), so that the clinical management can be tailored to individual patients.

Increased maternal BMI has a high prevalence in women developing GDM [approximately 65–75% are either overweight or obese (2, 3)] and was shown to be independently associated with pregnancy complications (such as fetal growth excess and adiposity) in the HAPO (Hyperglycaemia and Adverse Pregnancy Outcomes) study (4). Moreover, maternal obesity is associated with a higher level of insulin resistance, which is closely related to adverse pregnancy outcomes (like the development of large for gestational age offspring) (5, 6). In one of our previous studies we found that impaired insulin action at early pregnancy predicts the later requirement of glucose lowering medications, and therefore, an increased level of insulin resistance in mothers with overweight or obesity has possible impact on treatment modalities in those patients who develop GDM (7). While prepregnancy weight loss interventions are often not successful and are not recommended during pregnancy (8), a better understanding of the pathophysiological mechanisms linking pregestational BMI, the effectiveness of GDM treatment and adverse pregnancy outcomes could be used to further tailor the clinical pregnancy management of patients with obesity to their individual risk profile.

However, despite of major clinical importance, data on differences in treatment regimen and pregnancy outcomes in relation to the degree of maternal BMI in patients with GDM is sparsely available.

This study aims to evaluate differences in treatment modalities including use of insulin and metformin in relation to different degrees of obesity in pregnant women affected by GDM, taking into special consideration dosing regimens of basal insulin and rapid acting formulations. An analysis of pregnancy outcome and its association with maternal obesity is a further objective of this study.

## Methods

### Study Design and Participants

In this study, we included a total of 509 digitally registered patients with GDM, attending the pregnancy outpatient clinic at the Department of Obstetrics and Gynecology, Medical University of Vienna, between May 2015 and December 2017. Of a total of 617 originally registered patients during this period, 68 women were excluded because of preexisting diabetes, 10 patients were excluded because of pregestational diabetes, which was unrecognized before pregnancy, 25 women were excluded because of unclear GDM diagnosis (e.g. reported as GDM but negative OGTT results) and 5 patients were excluded because of missing pregestational BMI. Study participants self-reported their pregestational BMI (BMIPG) at their first visit (9, 10) and were categorized into women with normal weight (BMIPG <25 kg/m^2^), overweight (BMIPG ≥25 and <30 kg/m^2^) or obesity (BMIPG ≥30 kg/m^2^). GDM was diagnosed by use of a 75g 2h-OGTT according to the International Diabetes in Pregnancy Study Groups (IADPSG) recommendation (11). Glucose lowering medication (insulin and/or metformin) was started if glycemic targets were not achieved by lifestyle modification (i.e. if fasting or postprandial glucose levels exceeded 95 mg/dl or 140 mg/dl, according to international guidelines) (12). Data was assessed retrospectively; the following variables were extracted from a prospectively compiled database: age, height, weight before pregnancy, singleton or multiple pregnancy, OGTT glucose values (fasting, 60’ and 120’ after oral glucose load). Additionally, we assessed data on insulin treatment strategies: use and dose of metformin or insulin as well as details about insulin formulations (basal insulin such as intermediate or long-acting insulin analogues or rapid-acting insulin). All patients were treated with fixed insulin doses and the maximum doses per day were assessed. Moreover, neonatal outcome (birth weight, length and head circumference, gestational age at delivery (GAD), transfer to neonatal intensive care unit) and mode of delivery were assessed by referring to the medical history. Calculations of age and sex adjusted percentiles of the Austrian population were based on an analysis of the local growth standard curves. Small (SGA) and large for gestational age (LGA) were defined as bodyweight below the 10th and above the 90th percentile, respectively.

This study was approved by the local ethics committee and performed in accordance with the Declaration of Helsinki.

### Statistical Analysis

Continuous variables were summarized by mean ± standard deviation or as median and interquartile ranges (IQR) in case of skewed distribution and compared by analysis of variance or rank based inference, respectively. Dichotomous variables were summarized by counts and percentages, and compared by binomial logistic regression. Odds ratios (OR) and 95% confidence intervals (95%CI) were additionally calculated for binary outcomes. Fisher protected Least Significant Difference (LSD) tests were used for multiple comparisons (k=3 groups) to achieve a 95% coverage probability. Statistical analysis was performed with R (version 4.0.2) and contributing packages (13). A two-sided p-value of ≤0.05 was considered statistically significant.

## Results

**Figure 1:**
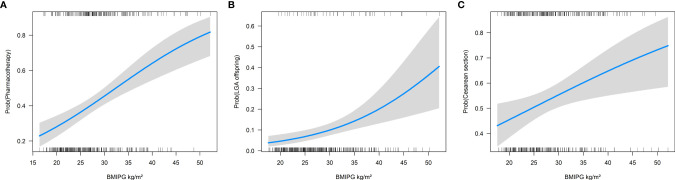
Association of pregestational BMI (BMIPG) with the probability of receiving pharmacotherapy **(A)**, for delivering large for gestational age (LGA) offspring **(B)** and having delivery by cesarean section **(C)**.

In **Table 1** main characteristics of the study sample are provided; categorized according to their pregestational BMI into GDM patients with normal weight (GDM-NW: n=200), overweight (GDM-OW: n=157) and obesity (GDM-OB: n=152). As compared to patients with normal weight, patients with overweight (mean difference, MD: 4.4 mg/dl, 95%CI 1.9 to 6.8, p<0.001) or obesity (MD: 6.7 mg/dl, 95%CI 4.3 to 9.2, p<0.001) had significantly higher fasting glucose concentrations, whereas OGTT glucose levels at 60’ and 120’ were comparable. Of note, obese women were diagnosed at an earlier gestational age. Higher pregestational BMI was significantly associated with the need of glucose lowering medications (OR: 1.08 for the increase of 1 kg/m² BMI, 95%CI 1.05 to 1.11, p<0.001), as visualized in **Figure 1A**. Insulin (basal or rapid acting) was more often prescribed for patients with overweight or obesity, whereby the latter showed the highest requirement of total insulin units per day (**Table 1**). There was no difference in the daily dose of basal insulin formulations between groups. However, GDM patients with obesity required a higher amount of rapid acting insulin as compared to normal weight patients. Metformin was more often used in patients with obesity who also required higher daily doses as compared to normal weight women. Moreover, a combination therapy with insulin and metformin was more often observed in gestational diabetic mothers with obesity. Glycemic control was evaluated in a subgroup of 220 patients at 38 (IQR: 37-39) weeks of gestation. Adequate glycemic control was protocolled in 74 (83%) normal weight, 49 (67%) overweight and 42 (72%) obese patients. Although there was a tendency that overweight and obese patients reached glycemic targets less often, the observed difference didn’t reach significance (p=0.052). Moreover, weight gain was assessed in 340 women around delivery (median 1.0, IQR: 0.0-2.0 weeks before delivery). It was found that normal weight patients had a weight gain of 13.2 ± 6.7 kg vs. 12.2 ± 6.1 kg in overweight patients (p=0.504). Obese patients gained 10.7 ± 7.6 kg which was significantly lower as compared to overweight (p=0.04) or obese women (p=0.006).

**Table 1:** Characteristics of the study sample.

	GDM-NW	GDM-OW	GDM-OB	p-value
	(n=200)	(n=157)	(n=152)	
Age (years)	31.6 ± 4.8	32.6 ± 4.9	32.0 ± 4.6	0.192
Height (cm)	164 ± 6.8	163 ± 6.8	164 ± 6.2	0.280
Weight, before pregnancy (kg)	59.0 ± 7.0	72.8 ± 6.7*	95.5 ± 15.3*^†^	<0.001
BMI, before pregnancy (kg/m^2^)	21.9 ± 2.0	27.4 ± 1.3*	35.6 ± 4.8*^†^	<0.001
Multiple pregnancy (%)	23 (11.4)	15 (9.6)	10 (6.6)	0.295
OGTT Glucose 0’ (mg/dl)	88.4 ± 12.2	92.8 ± 9.9*	95.2 ± 9.0*	<0.001
OGTT Glucose 60’ (mg/dl)	174.6 ± 33.2	179.1 ± 34.4	180.3 ± 33.4	0.336
OGTT Glucose 120’ (mg/dl)	135.2 ± 30.9	139.8 ± 31.4	138.8 ± 35.1	0.482
Gestational age at GDM diagnosis (weeks)	28.0 (26.0-29.5)	27.0 (25.0-30.0)	26.5 (23.0-28.0)*^†^	<0.001
Pharmacotherapy (Metformin and/or Insulin)	52 (26.0)	75 (47.8)*	84 (55.3)*	<0.001
Metformin only (%)	12 (6.0)	15 (9.6)	15 (9.9)	0.317
Metformin total (%)	16 (8.0)	19 (12.1)	27 (17.9)*	0.022
Metformin total (g/d)	1.50 (1.25-1.85)	1.50 (1.00-2.50)	2.50 (1.50-2.50)*	0.032
Insulin and Metformin (%)	4 (2.0)	4 (2.5)	12 (7.9)*^†^	0.016
Insulin BI or RAI total (%)	40 (20.0)	60 (38.2)*	69 (45.3)*	<0.001
Insulin BI or RAI total (IU/d)	24.0 (15.5-31.5)	30.0 (18.0-40.0)	40.0 (27.0-52.0)*^†^	0.019
Insulin BI total (%)	31 (15.5)	55 (35.0)*	63 (41.4)*	<0.001
Insulin BI total (IU/d)	12 (9-24)	12 (10-21)	16 (12-27)	0.122
Insulin RAI total (%)	24 (12.1)	31 (20.0)*	34 (22.4)*	0.026
Insulin RAI total (IU/d)	10 (4.5-15)	10 (5-19)	14 (10-26)*	0.029

Data are mean ± SD or median (IQR) and count (%) for patients with GDM and normal weight (GDM-NW) vs. overweight (GDM-OW) and obese patients (GDM-OB). BMI, body mass index; OGTT, oral glucose tolerance test; Insulin BI, basal insulin; insulin RAI, rapid acting insulin.

*p<0.05 vs. GDM-NW.

^†^p<0.05 vs. GDM-OW.

**Figure 2:**
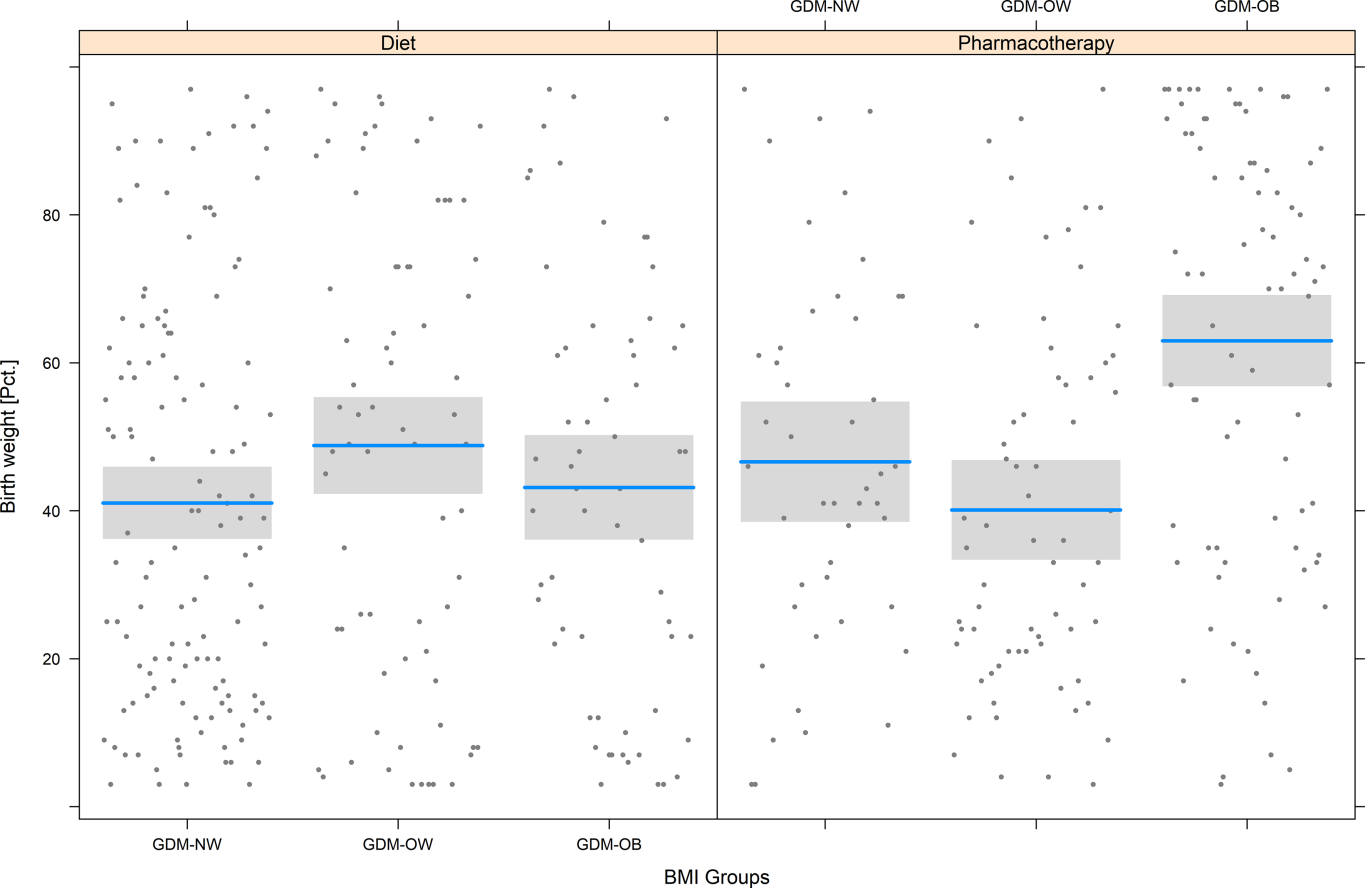
Birth weight percentiles in normal weight (GDM-NW), overweight (GDM-OW) or obese patients with GDM (GDM-OB), categorized further into patients who received glucose lowering pharmacotherapy (PT) or diet only. The grey box represents the interquartile range. The median is indicated by a blue line.

Assessment of pregnancy outcome data showed that pregestational BMI was associated with an increased risk of delivering LGA offspring (OR 1.09 for the increase of 1 kg/m² BMI, 95%CI 1.04 to 1.13, p<0.001, **Figure 1B**) as well as with an increased cesarean section rate (OR 1.04 for the increase of 1 kg/m² BMI, 95%CI 1.01 to 1.07, p<0.001, **Figure 1C**). Consequently, offspring of mothers with obesity showed higher birth weight percentiles as compared to normal weight and overweight patients as provided in **Table 2**. However, analysis of interaction indicated that the association of obesity status and birth weight percentiles of newborns was modified if pharmacotherapy was necessary in these patients (p<0.001 for the interaction between overweight or obesity status and requirement of pharmacotherapy for GDM). As compared to not medicated mothers with obesity (which showed mean birth weight of 43.2 ± 28.2 Pct.), birthweight percentiles were increased in patients with obesity who received glucose lowering medication (63.0 ± 28.8 Pct.), leading to a significant interaction (**Figure 2**). However, weight gain during pregnancy showed no significant association with LGA development in our study (p=0.591).

**Table 2:** Fetal biometry and pregnancy outcomes (multiple pregnancies and cases with missing pregnancy outcome data are excluded).

	GDM-NW	GDM-OW	GDM-OB	p-value
	(n=177)	(n=142)	(n=142)	
Cesarean Section	79 (44.6)	76 (53.5)	90 (63.4)*	0.004
Neonatal Intensive Care	13 (7.3)	10 (7.2)	11 (7.9)	0.976
GAD (weeks)	40 (39-41)	39 (39-41)	39 (39-40)*	0.028
Preterm Delivery (<37 GW)	17 (9.6)	11 (7.7)	9 (6.3)	0.558
Birth Weight (Percentile)	42.5 ± 27.3	44.6 ± 28.6	54.4 ± 30.1*^†^	<0.001
Birth Length (Percentile)	46.9 ± 26.3	48.2 ± 26.9	49.2 ± 28.7	0.758
Birth Head Circumference (Percentile)	42.2 ± 27.1	44.1 ± 28.9	53.3 ± 29.4*^†^	0.002
SGA (<10. Pct)	20 (11.5)	18 (13.0)	15 (10.6)	0.819
LGA (>90. Pct)	10 (5.7)	10 (7.2)	23 (16.3)*^†^	0.005

Data are mean ± SD or median (IQR) and count (%) for patients with GDM and normal weight (GDM-NW) vs. overweight (GDM-OW) and obese patients (GDM-OB). GAD, gestational age at delivery; SGA, small for gestational age offspring; LGA, large for gestational age offspring.

*p<0.05 vs. GDM-NW.

^†^p<0.05 vs. GDM-OW.

## Discussion

We aimed to assess characteristics of treatment modalities, neonatal outcome and delivery mode in women with GDM, categorized according to their pregestational BMI into patients with normal-weight, overweight or obesity. We found that pregestational BMI was significantly associated with an earlier diagnosis of GDM and increased requirement of glucose lowering medication, with mothers with obesity needing the highest amount of insulin units per day. This observation is in line with previous publications: Langer and co-workers found that mothers with overweight and obesity required a significantly higher total daily insulin dose (88 and 121 IU/d) as compared to normal weight GDM patients (69 IU/d) (14). Of note, the number of patients who received pharmacotherapy (69% vs. 41%) as well as the amount of required insulin was markedly higher as compared to our study. This might be explained by the use of different diagnosis criteria. There is some data suggesting that the IADPSG approach (which was used in our study) identifies more, but also less severe, cases of GDM as compared to other approaches [such as the Carpenter-Coustan criteria, which were used by Langer et al. (14)] (15). More recently, Machado et al. evaluated the impact of pregestational obesity on pregnancy outcomes in GDM pregnancies and observed that the need of pharmacological intervention was higher in patients with obesity (54%) as compared to patients with overweight (42%) or normal weight (29%) (16). This is comparable to our findings; however, the amount of insulin or metformin doses per day were not reported. In line with the above mentioned findings, another retrospective cohort study concluded that maternal BMI was the only predictor for the need of treatment with glucose lowering agents in patients with GDM (17).

The higher need of pharmacotherapy in patients with obesity may be explained by impaired insulin action, which is often prevalent already before conception. With advancing pregnancy insulin sensitivity decreases, leading to manifest hyperglycemia in patients who do not adequately compensate impaired insulin action by increased insulin release from the pancreatic β-cells (18–20). In this context, we observed increased fasting plasma glucose levels in patients with overweight and obesity at the time of GDM diagnosis, whereas glucose levels after oral glucose load were comparable between groups. Higher fasting glucose concentrations are pointing towards a primary defect in hepatic insulin sensitivity [as fasting glucose is mostly regulated by the liver (21)] in GDM patients with overweight or obesity and may be an underlying reason why basal insulin [which suppresses hepatic gluconeogenesis (22)] was more often used in those patients in our study. The higher requirement of rapid acting insulin may be due to additional defects in β-cell function.

Interestingly, GDM patients with obesity were more often treated with metformin [which also suppresses hepatic gluconeogenesis (23)] and required a higher dosage of this medication. We also observed that obesity resulted in an increased complementary treatment with insulin. This may possibly indicate an increased risk of secondary treatment failure of metformin in pregnant women with obesity. In this context, results from the MIG (Metformin in Gestational Diabetes) trial and other studies suggest that its use in pregnancy is save and has some advantages over insulin (especially being weight neutral and without risk of hypoglycemia) (24, 25). However, a significant treatment failure rate of metformin was already reported in the MIG trial, where 46% of the patients received supplemental insulin therapy (24). Other studies (26, 27) reported lower rates of metformin treatment failure. Previous research, aiming to assess the contributors to the observed failure rate of metformin monotherapy in pregnant women, concluded that the chance for responding to metformin was lower in patients with increased BMI (17, 28, 29) and higher fasting glucose levels (26, 28, 29). It is also noteworthy that large intervention studies failed to identify a protective effect of metformin on the risk of GDM, further suggesting that metformin is less effective in mothers with obesity (30, 31).

A number of studies indicated an increased risk of cesarean section delivery in mothers with obesity, potentially related to fetal adiposity (18). As shown in a secondary analysis of the HAPO study, the impact of increased maternal obesity on the risk of fetal growth excess is aggravated by hyperglycemia (32). This is in line with our results; however, our data further indicates that the well-known association between maternal and fetal adiposity is markedly increased in GDM patients with obesity who required glucose lowering therapy. There are two possible explanations for this observation: First; mothers with obesity have poor glycemic control despite receiving glucose lowering medication. Second; there is an underlying pathophysiologic factor (e.g. impaired insulin sensitivity) responsible for both, exceeded fetal growth as well as treatment failure of life-style modification, leading to the requirement of pharmacologic intervention in some patients. The latter theory is supported by observations suggesting that impaired insulin action in early pregnancy is per se contributing to fetal adiposity (due to hyperinsulinism and placental changes leading to increased nutrient supply to the offspring) but is also acting as a risk factor for the need of glucose lowering medication as described above (7, 18). We are not able to further elaborate those theories due to lack of detailed data about insulin sensitivity status in this retrospective study which is a limitation of this work. Impaired insulin sensitivity can possibly lead to increased maternal lipids, which can possibly explain the increased rates of LGA offspring in obese women (33, 34). We suggest further investigation of the possible interaction between the extent of insulin resistance (as well as other glucometabolic parameters in early pregnancy) and fetal development in patients with obesity. Also, the role of maternal body composition (especially the amount of visceral fat), lipid profiles and inflammatory markers should be further investigated to further disentangle the interaction between BMI, poor glycemic control and detrimental neonatal and maternal pregnancy outcomes in women with obesity.

In summary, we found that treatment modalities and outcomes in GDM pregnancies are strongly associated with the extent of maternal BMI and obesity status. Patients with obesity showed higher requirement of glucose lowering medication and were at higher risk of adverse pregnancy outcomes, such as cesarean section or LGA offspring. Our finding of markedly exceeded birth weight in infants of mothers with obesity who required glucose lowering medication is interesting and points towards an underlying role of early pregnancy insulin resistance, which interferes either with glycemic control or the development of fetal adiposity. It is crucial to further explore the underlying pathophysiologic mechanisms to optimize individual treatment approaches in these patients with particular risk for poor obstetric outcomes.

## Data Availability Statement

The raw data supporting the conclusions of this article will be made available by the authors, without undue reservation.

## Ethics Statement

This study was reviewed and approved by the Ethics Committee of the Medical University of Vienna, Borschkegasse 8b/E06, 1090 Vienna, Austria. The patients/participants provided their written informed consent to participate in this study.

## Author Contributions

CG conceived the study. Data assessment was performed by CM, AE, IR, DE, TL and CG. Calculations and data interpretation were performed by CG. Statistical analysis was performed by CG. CSG prepared tables and figures. The manuscript was written by CG and TL. CM, IR, DE, FG, KR, IH, EH critically revised the manuscript. All authors contributed to the article and approved the submitted version.

## Funding

The study was funded by a research grant from Sanofi to CSG.

## Conflict of Interest

The authors declare that the research was conducted in the absence of any commercial or financial relationships that could be construed as a potential conflict of interest.

## Publisher’s Note

All claims expressed in this article are solely those of the authors and do not necessarily represent those of their affiliated organizations, or those of the publisher, the editors and the reviewers. Any product that may be evaluated in this article, or claim that may be made by its manufacturer, is not guaranteed or endorsed by the publisher.
